# E-commerce readiness and training needs of small-scale dairy processors in Serbia: Understanding barriers and knowledge gaps

**DOI:** 10.1016/j.heliyon.2024.e27442

**Published:** 2024-03-07

**Authors:** Zorana Miloradovic, Jovana Kovacevic, Jelena Miocionovic, Ilija Djekic, Nemanja Kljajevic, Nada Smigic

**Affiliations:** a Department of Animal Source Food Technology, Faculty of Agriculture, University of Belgrade, Nemanjina 6, 11081, Belgrade, Serbia; b Food Innovation Center, 1207 NW Naito Parkway, Oregon State University, Portland, OR, 97209, USA; c Department of Food Safety and Quality Management, Faculty of Agriculture, University of Belgrade, Nemanjina 6, 11081, Belgrade, Serbia; d Institute of Molecular Genetics and Genetic Engineering, University of Belgrade, Vojvode Stepe 444a, 11042, Belgrade, Serbia; e Cheese Academy Association, Jurija Gagarina 182, 11070, Belgrade, Serbia

**Keywords:** Small scale dairy, Covid-19, e-commerce, Training needs assessment

## Abstract

The objective of this study was to identify the requirements needed for selling dairy products through e-commerce, as well as current gaps and challenges that exist for small scale dairy processors (SSDPs), and need to be addressed in order to comply with those requirements. A mixed method research design was used for training needs assessment. Qualitative (in-depth interview with 7 online platform representatives (OPRs)) and quantitative approach (survey questionnaire with 58 SSDPs) were conducted. Interview transcripts were coded and codes were grouped into seven themes. Hierarchical cluster analysis was applied to 146 answers from 58 SSDPs. They were divided into 4 clusters. Mean sums of responses between clusters were compared by Mann-Whitney *U* test. OPRs suggested that SSDPs should be provided with tools and resources to help them achieve food safety and quality targets, as well as practical knowledge and skills. They reported that it is crucial to find a solution for the cold chain transportation, for maintaining consistent product quality. Survey results showed that SSDPs use kitchen equipment (79.3%) and kitchen cleaning products (81.0%) for dairy processing. In total, 43.1% process raw milk and only 24.1% have product label on the package. Only members of cluster 3 and 4 sell their products online (73.7% and 90.0%, respectively), mostly using their own social media platforms (57.9% and 60.0%, respectively), transporting products to end buyers by themselves in hand refrigerators (47.4% and 70.0%, respectively). By analyzing the differences among clusters of SSDPs, trainings can be tailored to the characteristics and knowledge gaps of each group.

## Highlights

• Adequate packaging and proper labels are essential e-commerce requirements.
• Not a single survey participant sell product through a dedicated online only service.
• 43.1% of small scale dairy processors produce raw milk cheeses, mostly without labels.
• 75.9% of respondents would attend in-person trainings, 41.4% would try e-learning training.

## 1 Introduction

Serbia produces an average of 1.5 billion liters of raw milk annually, and a remarkable share (∼45%) of the total milk is processed by small-scale dairy processors (SSDPs), representing an important segment of the Serbian dairy industry [1]. Dairy products from SSDPs are typically sold on-site, at local markets, and in local restaurants. In small-scale manufacturing, the entire production from the raw material to the final product, including distribution, is usually managed by one or few family members. Manufacturing skills are mainly passed down from generation to generation [2], without formal training or knowledge to adequately assess the food safety risks associated with their products. Additionally, these processors face complex food safety rules and regulations that may be misinterpreted or difficult to understand. European Union food legislation, as well as Serbian legislation have provided some allowances for small scale food producers [[3], [4], [5],^37^]. These mainly pertain to the infrastructure, such as the facilities and equipment important in maintaining the hygienic process during the production. However, even with the modified requirements, understanding food safety risks and navigating the complexities of the regulations remain a significant challenge for small scale producers [6].

As a result of the COVID-19 pandemic, caused by SARS-CoV-2, many countries went into a lockdown in early 2020, affecting the food sectors worldwide [7], although the impact and long term consequences of these measures have not yet been fully quantified. In Serbia, only essential economic sectors remained open during this time, including agriculture and food production, while open markets and restaurants remained closed. Given their limited capacity to swiftly adapt to the evolving demands of the food market [8], many Serbian SSDPs found themselves bereft of options for selling their products during the lockdown. Since their products are perishable and have short shelf life, it was an additional challenge for them to move from traditional distribution channels (e.g., local markets, restaurants) to alternative markets in order to stay in business.

At the same time, the alternative markets, such as online distribution channels (e-commerce) showed a surge. Two types of markets emerged; dedicated online-only services and existing retailers with an online delivery option [9]. In January 2022 a comprehensive online search was conducted to acertain the number of SSDPs using e-commerce. It revealed that only 10 SSDPs in Serbia were included in specialized shops providing an online delivery option, while no SSDPs were listed in an online delivery database of major retailers in Serbia (data not published). However, a considerable number of SSDPs joined social media or website platforms that were established for marketing purposes and for facilitating direct connections between producers, often from rural areas, and consumers, mainly from urban areas [10]. This distribution channel is identified in the literature as a subset of e-commerce, and referred to as F-commerce or I-commerce, depending on a type of social media platform that is engaged, namely Facebook and Instagram [11].

COVID-19 pandemic has also impacted the demand side, with many people choosing to buy groceries online for the first time [9]. Results from our study in 2019, prior to the COVID-19 pandemic, showed that the majority (80%) of Serbian consumers purchased cheese at grocery store or supermarkets, followed by local open markets (50%), and direct sales from producers (25%), while only 0.31% reported buying dairy products through online channels [12]. Although online food delivery also existed in the pre-COVID-19 period, it was underutilized due to a lack of trust and familiarity with this service [9]. As a result of working from home, consumers’ habits shifted more to purchasing of foods with longer shelf life, and home cooking [13], with the online grocery retail becoming the major channel for food supply [14,15]. There is growing evidence that online grocery shopping is becoming increasingly accepted by consumers due to its convenience, availability of information, and increased selection [16]. It is also anticipated that the sustained expansion of e-commerce subsequent to the pandemic, will have a long-term positive influence on the overall environmental impact of the food system [17].

In addition, due to increased health and dietary concerns caused by the COVID-19 pandemic, a growing tendency to seek healthy, nutrient-rich foods has been observed among the consumers [15]. People have started to prioritize buying local foods, or food of regional/national origin [9]. With dairy products produced by SSDPs being perceived by Serbian consumers as highly valued products, superior in quality and health aspects when compared to the products produced by large-scale producers [1,12], it is imperative to improve the availability of such products. To do this effectively, it is essential to understand barriers and challenges that are presently preventing the expansion of the distribution and sale channels for SSDPs. The objective of this study was therefore to identify the requirements needed for selling dairy products through e-commerce, as well as current gaps and challenges that exist for SSDPs to comply with those requirements. This approach allowed us to identify the type of support needed by SSDPs, so that successful integration of these products into diverse distribution and sale platforms can be achieved, helping improve the sustainability of SSDPs and add resilience to the supply chain.

## 2 Material and methods

### 2.1 Overall approach

A mixed method research design, as proposed by Schoonenboom and Johnson [18] was used for training needs assessment guided (in terms of methodology) by previous literature [19,20]. The training needs assessment tool served as a means to systematically collect data on the current state of knowledge, practices, and challenges faced by SSDPs in the context of e-commerce readiness. Identification of these needs aimed to provide insights for the development of targeted training programs and support mechanisms, for enhancing the overall readiness and capabilities of small-scale dairy processors in the digital marketplace.

A mixed methods design is characterized by the combination of at least one qualitative and one quantitative research component for the broad purposes of breadth and depth of understanding and corroboration. According to Schoonenboom and Johnson [18], the research participants in qualitative and quantitative part of the research could be similar or different. Furthermore, in each mixed method there is the point of integration at which the qualitative and quantitative components are brought together. In this particular study participants are different. The results from in-depth interviews conducted with OPRs, have been used to improve a questionnaire for SSDPs, which is defined as *instrument development* point of integration. The results from both strands were performed independently, brought together and the aspects of the same mixed research questions were addressed.

The study was determined exempt from the need for approval by the Institutional Review Board at Oregon State University (study # HE-2022-89; date of exemption: December 15, 2022). All researchers responsible for realization of in-depth interviews and survey completed the Human Research Protection Training provided by the Office for Human Research Protections (Department of Health & Human Services, Washington D.C., USA).

### 2.2 Qualitative training needs assessment

#### 2.2.1 Development of the interview guide

Interview questions were developed following discussions with key stakeholders and researchers (n = 17). The stakeholders included representatives from the Serbian Chamber of Commerce, USAID mission in Serbia, Oregon State University, Ministry of Agriculture and various SSDP associations. They were selected based on their experience working with SSDPs, and their knowledge and expertise regarding training programs for small scale producers, legal requirements for dairy processing in small scale facilities and the operation of e-commerce.

A general flow and specific questions (n = 12) for in-depth interview with Online Platform Representatives (OPRs) were developed. A short questionnaire including the demographics (i.e., gender, age, education and the employment information) was also included. The interview questions were reviewed by an expert panel of food science and food safety specialist and Extension educators (5) and revised for clarity and flow.

#### 2.2.2 Recruitment of OPR participants and interview realization

OPRs were identified through searches on social media accounts and websites. Additionally, given the relatively small size of the food e-commerce platforms sector in Serbia, word-of-mouth referrals from within the community were utilized. Contact was established through phone or email communication. Interviewees consisted of those willing to participate in face-to-face or online meetings, allocating a maximum of 1 h for the interview sessions. Seven OPRs were recruited, including four males and three females. They included representatives from the international Dedicated Online-Only Services (DOOS; n = 2), Retailers with an Online Delivery Option (RODO; n = 2), and representatives from the Associations with Online Platforms (social media and/or websites) for SSDP products promotion and facilitating direct connections between small scale producers and consumers (AOP; n = 3). In-depth interviews were conducted in the period of December 2022 to February 2023, five of them face-to-face and two of them (AOP representatives) online.

### 2.3 Quantitative training needs assessment

#### 2.3.1 Questionnaire development

Based on the information collected as part of the qualitative training needs assessment, the questionnaire was developed targeting SSDPs in Serbia. Single choice (yes/no), multiple choice and checklists were included. The questionnaire was piloted on dairy experts in Serbia and U.S. (n = 5) and on a sub-set of targeted audience (n = 5) to check for clarity and appropriateness of questions before being disseminated more widely to SSDPs. Following expert review, the final questionnaire included 43 questions and 167 answer options. Questions were divided into four sections: 1) dairy production systems, processing practices and type of products (18 questions/67 answer options); 2) marketing and distribution practices (10 questions/45 answer options); 3) digital proficiencies and e-commerce perspectives (9 questions/35 answer options; 4) training preferences (6 questions/20 answer options). Single choice and multiple choice questions were included.

#### 2.3.2 Recruitment of SSDPs participants and survey administration

Participant recruitment was multifold. Local Chambers of Commerce and non-governmental organizations provided personal contacts of SSDPs. Social media accounts and websites were searched for SSDPs, and recruitment information was shared with each identified business. Additionally, potential survey participants were recruited through word-of-mouth, particularly at the Balkan Cheese Festival. All participants had to be at least 18 years old, be owners or members of an agricultural household in Serbia, and produce cow, sheep, or goat milk on their farm.

**Fig. 1:**
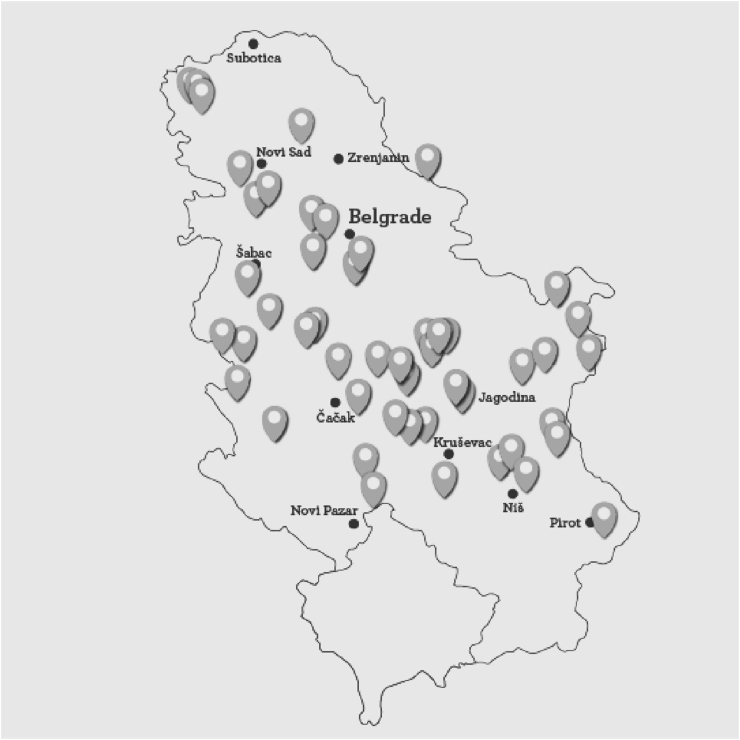
Geoghraphical locations of 50 villages in Serbia visited for the survey realization.

Survey participants were those who were willing to meet with researchers in person for the purpose of the survey. They were interviewed preferably, but not exclusively, at their farms and/or production facilities with the goal to cover the widest possible territory within Serbia (Fig. 1). In each instance, researchers verbally posed questions along with answer options and personally filled the questionnaire based on the provided answers. In total, 58 survey responses, encompassing SSDPs from 50 villages across Serbia, were collected from February to May 2023.

### 2.4 Data analysis

Each in-depth interview was audio recorded, with written consent from participants. Interviews were transcribed by one trained researcher and reviewed independently by a different researcher. Two researchers were involved in coding the transcript of the interview – a coder and a checker. A coder looked closely at the transcript, extracted information and created a codebook. A checker reviewed the codebook and together with a coder developed the final version of the codebook (Supplementary material). Codes were grouped into seven themes for analysis purposes.

Questionnaire answer options were organized in the following manner. For single choice yes and no questions, yes and no answers were assigned values of 1 and 0, respectively. For multiple-choice questions and checklists, all answers selected by participants were assigned a score of 1, while those not selected were scored as 0. A total of 167 survey responses were analyzed. Data were presented as sums and percentage (in brackets) of participants that provided positive answers (Table 1, Table 2, Table 3, Table 4).

**Table 1:** Survey participants’ dairy production systems, processing practices and type of products accross clusters.

Questions and answers offered to respondents	% of Participants (Frequency of Selected Answers)					
	Total % (N)		Cluster 1 % (N)	Cluster 2 % (N)	Cluster 3 % (N)	Cluster 4 % (N)
Percentage (number) of respondents	100.0 (58)		29.3 (17)	20.7 (12)	32.7 (19)	17.2 (10)
**1. Registered as an agricultural household (yes/no)**	98.3 (57)		100.0 (17)	100.0 (12)	100.0 (19)	90.0 (9)
**2. Registered under the Veterinary Directorate**rowhead						
2.1. Yes	37.9 (22)		17.6 (3)^ab^	8.3 (1)^a^	57.9 (11)^b^	70.0 (7)^bc^
2.2. No, we are currently in the registration process	1.7 (1)		0.0 (0)	0.0 (0)	5.3 (1)	0.0 (0)
2.3. No, we are planning to apply for registration	10.3 (6)		0.0 (0)	16.7 (2)	10.5 (2)	20.0 (2)
2.4. No	50.0 (29)		82.4 (14)^ab^	75.0 (9)^a^	26.3 (5)^b^	10.0 (1)^bc^
**3. Inspected by the Veterinary Directorate?**rowhead						
3.1. Yes	24.1 (14)		17.6 (3)^a^	8.3 (1)^a^	15.8 (3)^a^	70.0 (7)^b^
3.2. No, we are currently in the process of approval	0.0 (0)		0.0 (0)	0.0 (0)	0.0 (0)	0.0 (0)
3.3. No, we are planning to apply for approval	20.7 (12)		5.9 (1)	8.3 (1)	42.1 (8)	20.0 (2)
3.4. No	55.2 (32)		76.5 (13)^a^	83.3 (10)^a^	42.1 (8)^b^	10.0 (1)^b^
**4. Have membership in association or cooperative (yes/no)**	31.0 (18)		0.0 (0)^a^	25.0 (3)^ab^	42.1 (8)^b^	70.0 (7)^b^
**5. Milk products produced (multiple choices possible)**rowhead						
5.1. White brined cheese	81.0 (47)		94.1 (16)	58.3 (7)	94.7 (18)	60.0 (6)
5.2. Cream cheese, fresh cheese (Quark type)	36.2 (21)		11.8 (2)^b^	58.3 (7)^a^	47.4 (9)^ab^	30.0 (3)^ab^
5.3. Whey cheese	8.6 (5)		0.0 (0)	0.0 (0)	21.1 (4)	10.0 (1)
5.4. Whey	24.1 (14)		35.3 (6)	0.0 (0)	36.8 (7)	10.0 (1)
5.5. Semi hard/hard cheese	46.5 (27)		29.4 (5)^a^	0.0 (0)^a^	68.4 (13)^b^	90.0 (9)^b^
5.6. Kajmak	37.9 (22)		82.4 (14)^b^	0.0 (0)^a^	42.1 (8)^a^	0.0 (0)^a^
5.7. Other	12.1 (7)		5.9 (1)	0.0 (0)	10.5 (2)	40.0 (4)
**6. Sources of raw milk**rowhead						
6.1. Own farm only	81.0 (47)		94.1 (16)	91.7 (11)	78.9 (15)	50.0 (5)
6.2. Own farm, and sourced from others (e.g., neighbors)	10.3 (6)		5.9 (1)	8.3 (1)	10.5 (2)	20.0 (2)
6.3. Sourced from others (e.g., neighbors)	8.6 (5)		0.0 (0)	0.0 (0)	10.5 (2)	30.0 (3)
**7. Volume of processed milk in compliance with the regulation (yes/no)**	32.7 (19)		29.4 (5)^ab^	8.3 (1)^a^	31.6 (6)^ab^	70.0 (7)^b^
**8. Milking of animals on the farm**rowhead						
8.1. Manually	19.0 (11)		17.6 (3)	16.7 (2)	26.3 (5)	10.0 (1)
8.2. Mechanically	70.7 (41)		70.6 (12)	83.3 (10)	68.4 (13)	60.0 (6)
8.3. Combined, manually/mechanically	3.4 (2)		11.8 (2)	0.0 (0)	0.0 (0)	0.0 (0)
**9. Where raw milk is processed (multiple choices possible)**rowhead						
9.1. In the kitchen	20.7 (12)	41.2 (7)		16.7 (2)	15.8 (3)	0.0 (0)
9.2. In a separate room of the house	39.6 (23)	23.5 (4)		58.3 (7)	47.4 (9)	30.0 (3)
9.3. In a separate building on the same property as the house	39.6 (23)	35.3 (6)		25.0 (3)	36.8 (7)	70.0 (7)
**10. Type of milk used to make cheese (multiple choices possible)**rowhead						
10.1. Boiled/thermally treated milk	72.4 (42)	100.0 (17)^b^		0.0 (0)^a^	84.2 (16)^b^	90.0 (9)^b^
10.2. Uncooked/raw milk	43.1 (25)	0.0 (0)^b^		100.0 (12)^a^	52.6 (10)^b^	30.0 (3)^b^
**11. Ingredients used in the production of cheese (multiple choices possible)**rowhead						
11.1. Liquid rennet	87.9 (51)	100.0 (17)^a^		100.0 (12)^a^	100.0 (19)^a^	40.0 (4)^b^
11.2. Rennet powder	12.1 (7)	0.0 (0)^a^		0.0 (0)^a^	10.5 (2)^ab^	50.0 (5)^b^
11.3. Starter culture	10.3 (6)	0.0 (0)^a^		0.0 (0)^a^	5.3 (1)^a^	50.0 (5)^b^
11.4. Acids (e.g., lactic, citric, acetic)	13.8 (8)	0.0 (0)		0.0 (0)	36.8 (7)	10.0 (1)
11.5. Food colors	1.7 (1)	0.0 (0)		0.0 (0)	0.0 (0)	10.0 (1)
11.6. Plant supplements (e.g., rosemary, paprika, cranberries)	29.3 (17)	5.9 (1)^a^		0.0 (0)^a^	47.4 (9)^b^	70.0 (7)^b^
**12. Equipment used in milk processing (multiple choices possible)**rowhead						
12.1. Pot, kitchen table, plastic dishes, stove	79.3 (46)	100.0 (17)^a^		100.0 (12)^a^	73.7 (14)^ab^	30.0 (3)^b^
12.2. Cooling vat	31.0 (18)	5.9 (1)^a^		16.7 (2)^a^	31.6 (6)^a^	90.0 (9)^b^
12.3. Cheese making vat	27.6 (16)	5.9 (1)^a^		0.0 (0)^a^	26.3 (5)^a^	100.0 (10)^b^
12.4. Cheese press	22.4 (13)	5.9 (1)^a^		0.0 (0)^a^	21.1 (4)^a^	80.0 (8)^b^
12.5. Cheese molds	36.2 (21)	11.8 (2)^a^		8.3 (1)^a^	57.9 (11)^b^	70.0 (7)^b^
12.6. Cheese table	31.0 (18)	29.4 (5)^a^		25.0 (3)^a^	10.5 (2)^a^	80.0 (8)^b^
**13. Parameters that are controlled during milk processing (multiple choices possible)**rowhead						
13.1. Temperature	58.6 (34)	52.9 (9)^ab^		16.7 (2)^a^	68.4 (13)^bc^	100.0 (10)^c^
13.2. Time	39.6 (23)	5.9 (1)^a^		33.3 (4)^ab^	52.6 (10)^b^	80.0 (8)^b^
13.3. Acidity	20.7 (12)	0.0 (0)^a^		0.0 (0)^a^	15.8 (3)^a^	90.0 (9)^b^
**14. Cleaning and disinfecting agents used during milk processing (multiple choices possible)**rowhead						
14.1. Kitchen cleaning/disinfecting products	81.0 (47)	94.1 (16)		83.3 (10)	73.7 (14)	70.0 (7)
14.2. Dairy industry cleaning/disinfecting products	20.7 (12)	5.9 (1)		16.7 (2)	15.8 (3)	60.0 (6)
14.3. Other	5.2 (3)	0.0 (0)		0.0 (0)	10.5 (2)	10.0 (1)
**15. Packaging of dairy products (multiple choices possible)**rowhead						
15.1. Plastic, disposable box	58.6 (34)	82.4 (14)^b^		33.3 (4)^a^	78.9 (15)^b^	10.0 (1)^a^
15.2. Plastic, sealed box	6.9 (4)	17.6 (3)		8.3 (1)	0.0 (0)	0.0 (0)
15.3. Plastic bag	36.2 (21)	35.3 (6)^b^		83.3 (10)^a^	26.3 (5)^b^	0.0 (0)^b^
15.4. Vacuum package	43.1 (25)	5.9 (1)^a^		16.7 (2)^a^	63.2 (12)^b^	100.0 (10)^b^
15.5. Wax	1.7 (1)	0.0 (0)		0.0 (0)	0.0 (0)	10.0 (1)
15.6. Other	1.7 (1)	0.0 (0)		8.3 (1)	0.0 (0)	0.0 (0)
**16. Have product label on the package (yes/no)**	24.1 (14)	0.0 (0)^a^		0.0 (0)^a^	21.1 (4)^a^	100.0 (10)^b^
**17. Information included on the label (multiple choices possible)**rowhead						
17.1. Product name	24.1 (14)	0.0 (0)^a^		0.0 (0)^a^	21.1 (4)^a^	100.0 (10)^b^
17.2. List of ingredients	17.2 (10)	0.0 (0)^a^		0.0 (0)^a^	0.0 (0)^a^	100.0 (10)^b^
17.3. Production date	13.8 (8)	0.0 (0)^a^		0.0 (0)^a^	10.5 (2)^a^	60.0 (6)^b^
17.4. Expiration date	17.2 (10)	0.0 (0)^a^		0.0 (0)^a^	5.3 (1)^a^	90.0 (9)^b^
17.5. Storage conditions	19.0 (11)	0.0 (0)^a^		0.0 (0)^a^	10.5 (2)^a^	90.0 (9)^b^
17.6. Manufacturer's name and address	22.4 (13)	0.0 (0)^a^		0.0 (0)^a^	15.8 (3)^a^	100.0 (10)^b^
17.7. Registration number of the object	10.3 (6)	0.0 (0)^a^		0.0 (0)^a^	0.0 (0)^a^	60.0 (6)^b^
17.8. Weight/mass	15.5 (9)	0.0 (0)^a^		0.0 (0)^a^	5.3 (1)^a^	80.0 (8)^b^
17.9. Logo/Some special image/graphic	17.2 (10)	0.0 (0)^a^		0.0 (0)^a^	5.3 (1)^a^	90.0 (9)^b^
17.10. Special labels (organic, protection of origin, etc.)	3.4 (2)	0.0 (0)		0.0 (0)	0.0 (0)	20.0 (2)
**18. Keeping records during milk processing (multiple choices possible)**rowhead						
18.1. Dairy processing journal	22.4 (13)	0.0 (0)^a^		0.0 (0)^a^	31.6 (6)^ab^	70.0 (7)^b^
18.2. Records on the procurement of materials and ingredients	27.6 (16)	11.8 (2)^a^		0.0 (0)^a^	36.8 (7)^ab^	70.0 (7)^b^
18.3. Record of sold goods	43.1 (25)	29.4 (5)^a^		33.3 (4)^ab^	42.1 (8)^ab^	80.0 (8)^b^

Values in the table represent percentages and number - % (N) of respondents giving the positive answer; a–c Values in the superscript within a row with different letters were significantly different (p < 0.05) within clusters, as determined by Mann– Whitney *U* test.

**Table 2:** Survey participants’ marketing and distribution practices across clusters.

Qestions and answers offered to respondents	% of Participants (Frequency of Selected Answers)				
	Total % (N)	Cluster 1 % (N)	Cluster 2 % (N)	Cluster 3 % (N)	Cluster 4 % (N)
**1. Where do you sell your products (multiple choices possible)?**					
1.1. Home-based	82.7 (48)	94.1 (16)	75.0 (9)	84.2 (16)	70.0 (7)
1.2. Own catering facility/rural tourism	5.2 (3)	0.0 (0)	0.0 (0)	5.3 (1)	20.0 (2)
1.3. Open market	37.9 (22)	23.5 (4)^a^	41.7 (5)^ab^	63.2 (12)^b^	10.0 (1)^a^
1.4. Food fair/festival	24.1 (14)	0.0 (0)^a^	0.0 (0)^a^	26.3 (5)^a^	90.0 (9)^b^
1.5. Retail store	13.8 (8)	0.0 (0)^a^	0.0 (0)^a^	5.3 (1)^a^	70.0 (7)^b^
1.6. Via internet/online platform	27.6 (16)	0.0 (0)^a^	0.0 (0)^a^	47.4 (9)^b^	70.0 (7)^b^
1.7. Restaurant, hotel, catering	20.7 (12)	11.8 (2)	33.3 (4)	10.5 (2)	40.0 (4)
**2. Have you ever returned unsold products back to the facility? (yes/no)**	36.2 (21)	23.5 (4)	25.0 (3)	52.6 (8)	60.0 (6)
**3. Dealing with the unsold product within the expiration date (multiple choices possible)**					
3.1. We eat it	32.7 (19)	23.5 (4)	16.7 (2)	47.4 (9)	40.0 (4)
3.2. We sell it as soon as possible	20.7 (12)	5.9 (1)	25.0 (3)	31.6 (6)	20.0 (2)
3.3. We throw it in the garbage	8.6 (5)	0.0 (0)	8.3 (1)	5.3 (1)	30.0 (3)
3.4. Pigs eat it	3.4 (2)	0.0 (0)	0.0 (0)	0.0 (0)	20.0 (2)
3.5. Other	1.7 (1)	0.0 (0)	0.0 (0)	5.3 (1)	0.0 (0)
**4. The most important thing for selling dairy products (multiple choices possible)**					
4.1. Product appearance	50.0 (29)	35.3 (6)	33.3 (4)	68.4 (13)	60.0 (6)
4.2. The taste of the product	87.9 (51)	100.0 (17)	66.7 (8)	89.5 (17)	90.0 (9)
4.3. The price	44.8 (26)	35.3 (6)	25.0 (3)	47.4 (9)	80.0 (8)
4.4. Packaging	34.8 (20)	5.9 (1)^a^	16.7 (2)^ab^	52.6 (10)^b^	70.0 (7)^b^
4.5. Communication with customers	48.3 (28)	47.1 (8)	16.7 (2)	57.9 (11)	70.0 (7)
4.6. Other	3.4 (2)	0.0 (0)	0.0 (0)	5.3 (1)	10.0 (1)
**5. Reaction regarding complains about the quality of the product (multiple choices possible)**					
5.1. I would apologize	46.5 (27)	64.7 (11)	33.3 (4)	36.8 (7)	50.0 (5)
5.2. I would give a discount on the next purchase	10.3 (6)	0.0 (0)	8.3 (1)	21.1 (4)	10.0 (1)
5.3. I would offer a similar product for free	24.1 (14)	11.8 (2)	25.0 (3)	21.1 (4)	50.0 (5)
5.4. I would return customers money	44.8 (26)	47.1 (8)^b^	0.0 (0)^a^	68.4 (13)^b^	50.0 (5)^b^
5.5. Nothing, I can't please everyone	22.4 (13)	5.9 (1)	41.7 (5)	21.1 (4)	30.0 (3)
5.6. Other	3.4 (2)	5.9 (1)	0.0 (0)	5.3 (1)	0.0 (0)
**6. Familiarity with the term “food fraud” (yes/no)**	79.3 (46)	82.4 (14)	66.7 (8)	89.5 (17)	70.0 (7)
**7. How many instances of food fraud can you remember?**					
7.1. Example 1	58.6 (34)	76.5 (13)^a^	58.3 (7)^ab^	57.9 (11)^ab^	30.0 (3)^b^
7.2. Example 2	18.9 (11)	23.5 (4)	33.3 (4)	15.8 (3)	0.0 (0)
7.3. Example 3	5.2 (3)	5.9 (1)	8.3 (1)	5.3 (1)	0.0 (0)
**8. Food fraud is (multiple choices possible):**					
8.1. Selling milk from cows that were given antibiotics	55.2 (32)	52.9 (9)	41.7 (5)	63.2 (12)	60.0 (6)
8.2. When selling a neighbors' product claiming it's ours	58.6 (34)	47.1 (8)	58.3 (7)	52.6 (10)	90.0 (9)
8.3. When milk is diluted with water	63.8 (37)	47.1 (8)	66.7 (8)	78.9 (15)	60.0 (6)
8.4. When the product is sold as fresh, and it was made 10 days ago	50.0 (29)	29.4 (5)	50.0 (6)	57.9 (11)	70.0 (7)
8.5. Other	1.7 (1)	5.9 (1)	0.0 (0)	0.0 (0)	0.0 (0)
**9. Plans for the future (multiple choices possible)**					
9.1. Expanding the assortment of products	15.5 (9)	11.8 (2)	16.7 (2)	21.1 (4)	10.0 (1)
9.2. Expanding the capacity of milk production	25.9 (15)	0.0 (0)^a^	41.7 (5)^ab^	42.1 (8)^b^	20.0 (2)^ab^
9.3. Investing in the equipment	37.9 (22)	17.6 (3)	58.3 (7)	47.4 (9)	30.0 (3)
9.4. Cessation of production	6.9 (4)	11.8 (2)	8.3 (1)	5.3 (1)	0.0 (0)
9.5. Other	8.6 (5)	11.8 (2)	0.0 (0)	10.5 (2)	10.0 (1)
**10. Major challenges (multiple choices possible)**					
10.1. Product spoilage	10.3 (6)	5.9 (1)	0.0 (0)	15.8 (3)	20.0 (2)
10.2. Variations in product quality	15.5 (9)	5.9 (1)	16.7 (2)	21.1 (4)	30.0 (3)
10.3. Low price of the product	20.7 (12)	23.5 (4)^ab^	50.0 (6)^a^	0.0 (0)^b^	20.0 (2)^ab^
10.4. Lack of a market	17.2 (10)	0.0 (0)^a^	8.3 (1)^ab^	42.1 (8)^b^	10.0 (1)^ab^
10.5. Lack of knowledge	29.3 (17)	11.8 (2)	41.7 (5)	31.6 (6)	40.0 (4)
10.6. Other	5.2 (3)	11.8 (2)	0.0 (0)	5.3 (1)	0.0 (0)

Values in the table represent percentages and number - %(N) of respondents giving the positive answer; a–c Values in the superscript within a row with different letters were significantly different (p < 0.05) within clusters, as determined by Mann– Whitney *U* test.

**Table 3:** Survey participants’ digital proficiencies and e-commerce perspectives across clusters.

Qestions and answers offered to respondents	% of Participants (Frequency of Selected Answers)				
	Total % (N)	Cluster 1 % (N)	Cluster 2 % (N)	Cluster 3 % (N)	Cluster 4 % (N)
**1. Have an e-mail account (yes/no)**	79.3 (46)	47.1 (8)^a^	75.0 (9)^ab^	100.0 (19)^b^	100.0 (10)^b^
**2. If you do, how often do you check your e-mail?**					
2.1. At least once a day	51.7 (30)	23.5 (4)^a^	41.7 (5)^ab^	63.2 (12)^b^	90.0 (9)^b^
2.2. Once a week	8.6 (5)	11.8 (2)	8.3 (1)	5.3 (1)	10.0 (1)
2.3. When it comes to my mind, rarely	18.9 (11)	11.8 (2)	25.0 (3)	31.6 (6)	0.0 (0)
**3. Do you have Facebook/Instagram profile/account? (yes/no)**	86.2 (50)	58.8 (10)^a^	91.7 (11)^ab^	100.0 (19)^b^	100.0 (10)^b^
**4. If you have a Facebook/Instagram profile, how often do you check them?**					
4.1. At least once a day	65.5 (38)	58.8 (10)	91.7 (11)	94.7 (18)	90.0 (9)
4.2. Once a week	1.7 (1)	0.0 (0)	0.0 (0)	5.3 (1)	0.0 (0)
4.3. When it comes to my mind, rarely	0.0 (0)	0.0 (0)	0.0 (0)	0.0 (0)	10.0 (1)
**5. Do you use the Internet to sell your products?**					
5.1. Yes	39.6 (23)	0.0 (0)^a^	0.0 (0)^a^	73.7 (14)^b^	90.0 (9)^b^
5.2. No, but I have planned to use it	10.3 (6)	11.8 (2)	0.0 (0)	15.8 (3)	10.0 (1)
5.3. No	46.5 (27)	88.2 (15)^a^	100,0 (12)^a^	10.5 (2)^b^	0.0 (0)^b^
**6. If you use the Internet to sell your products, how do you do it (multiple choices possible)?**					
6.1. I have my own website	6.9 (4)	0.0 (0)	0.0 (0)	5.3 (1)	30.0 (3)
6.2. I have my Facebook/Instagram profile	31.0 (18)	0.0 (0)^a^	0.0 (0)^a^	57.9 (11)^b^	60.0 (6)^b^
6.3. I sell through association with an online platform (AOP)	13.8 (8)	0.0 (0)	0.0 (0)	36.8 (7)	10.0 (1)
6.4. I sell through the retail with an online sales option (RODO)	8.6 (5)	0.0 (0)	0.0 (0)	10.5 (2)	30.0 (3)
6.5. I sell through dedicated online only service (DOOS)	0.0 (0)	0.0 (0)	0.0 (0)	0.0 (0)	0.0 (0)
**7. If you use the Internet to sell your products, how do you deliver them (multiple choices possible)?**					
7.1. By own vehicle, in the trunk	10.3 (6)	0.0 (0)	0.0 (0)	31.6 (6)	0.0 (0)
7.2. By own vehicle, in hand-held refrigerators	27.6 (16)	0.0 (0)^a^	0.0 (0)^a^	47.4 (9)^b^	70.0 (7)^b^
7.3. By own vehicle, which has a cooling chamber	3.4 (2)	0.0 (0)	0.0 (0)	5.3 (1)	10.0 (1)
7.4. I use express mail services	17.2 (10)	0.0 (0)	0.0 (0)	31.6 (6)	40.0 (4)
7.5. I am hiring a transporter with a refrigerated vehicle	8.6 (5)	0.0 (0)	0.0 (0)	21.1 (4)	10.0 (1)
**8. In your opinion, what are the advantages of selling on the Internet (multiple choices possible)?**					
8.1. I can sell goods faster	29.3 (17)	29.4 (5)^ab^	0.0 (0)^a^	47.4 (9)^b^	30.0 (3)^ab^
8.2. I can sell more goods	37.9 (22)	29.4 (5)	25.0 (3)	63.2 (12)	50.0 (5)
8.3. I can sell all over Serbia	27.6 (16)	0.0 (0)^a^	25.0 (3)^ab^	36.8 (7)^ab^	60.0 (6)^b^
8.4. I can communicate with customers faster	8.6 (5)	0.0 (0)	0.0 (0)	10.5 (2)	30.0 (3)
8.5. I can charge immediately	1.7 (1)	0.0 (0)	0.0 (0)	0.0 (0)	10.0 (1)
8.6. I can charge more for products	18.9 (11)	23.5 (4)	16.7 (2)	15.8 (3)	20.0 (2)
8.7. Other	3.4 (2)	0.0 (0)	0.0 (0)	10.5 (2)	0.0 (0)
**9. In your opinion, what are the disadvantages of e-commerce (multiple choices possible)?**					
9.1. I have no direct communication with the customer	29.3 (17)	29.4 (5)	8.3 (1)	31.6 (6)	50.0 (5)
9.2. I don't have enough knowledge	10.3 (6)	29.4 (5)	8.3 (1)	0.0 (0)	0.0 (0)
9.3. I can't be on the Internet all the time	22.4 (13)	41.2 (7)	8.3 (1)	21.1 (4)	10.0 (1)
9.4. I don't like payment via account, I like cash	1.7 (1)	0.0 (0)	8.3 (1)	0.0 (0)	0.0 (0)
9.5. I don't want others to make money off of me	5.2 (3)	5.9 (1)	8.3 (1)	5.3 (1)	0.0 (0)
9.6. Additional time and additional organization are necessary	34.5 (20)	47.1 (8)	16.7 (2)	36.8 (7)	30.0 (3)
9.7. Other	10.3 (6)	11.8 (2)	8.3 (1)	15.8 (3)	0.0 (0)

Values in the table represent percentages and number - %(N) of respondents giving the positive answer; a–c Values in the superscript within a row with different letters were significantly different (p < 0.05) within clusters, as determined by Mann– Whitney *U* test.

**Table 4:** Survey participants’ training preferences across clusters.

Qestions and answers offered to respondents	% of Participants (Frequency of Selected Answers)				
	Total % (N)	Cluster 1 % (N)	Cluster 2 % (N)	Cluster 3 % (N)	Cluster 4 % (N)
**1. Have you attended any training related to milk processing and business In the last five years? (yes/no)**	19.0 (11)	0.0 (0)	8.3 (1)	26.3 (5)	50.0 (5)
**2. Interest in the training on milk processing**					
2.1. Yes	62.1 (36)	35.3 (6)^a^	58.3 (7)^ab^	84.2 (16)^b^	70.0 (7)^ab^
2.2. Maybe	31.0 (18)	52.9 (9)^a^	33.3 (4)^ab^	10.5 (2)^b^	30.0 (3)^ab^
2.3. No	6.9 (4)	11.8 (2)	8.3 (1)	5.3 (1)	0.0 (0)
**3. Choice of the way of conducting the training (multiple choices possible)**					
3.1. Face-to-face	75.9 (44)	76.5 (13)	75.0 (9)	73.7 (14)	80.0 (8)
3.2. Via internet/e-learning training	41.4 (24)	23.5 (4)	25.0 (3)	57.9 (11)	60.0 (6)
3.3. Other	0.0 (0)	0.0 (0)	0.0 (0)	0.0 (0)	0.0 (0)
**4. Choice of the training topics (multiple choices possible)**					
4.1. Legal requirements and regulations	55.2 (32)	47.1 (8)	66.7 (8)	57.9 (11)	50.0 (5)
4.2. Communication with customers	34.5 (20)	35.3 (6)	33.3 (4)	36.8 (7)	30.0 (3)
4.3. Improvement of dairy product technology	79.3 (46)	70.6 (12)	83.3 (10)	94.7 (18)	60.0 (6)
4.4. Production, packaging and product marketing	60.3 (35)	64.7 (11)	50.0 (6)	57.9 (11)	70.0 (7)
4.5. Food hygiene conditions during production and transport	48.3 (28)	52.9 (9)	41.7 (5)	52.6 (10)	40.0 (4)
4.6. Product labeling	48.3 (28)	41.2 (7)	66.7 (8)	52.6 (10)	30.0 (3)
4.7. Other	5.2 (3)	11.8 (2)	8.3 (1)	0.0 (0)	0.0 (0)
**5. Have you ever watched content related to milk processing on the Internet (multiple choices possible)?**					
5.1. Yes, I attended online training	0.0 (0)	0.0 (0)	0.0 (0)	0.0 (0)	0.0 (0)
5.2. Yes, I watched various you-tube contents	75.9 (44)	64.7 (11)	75.0 (9)	89.5 (17)	70.0 (7)
5.3. No	24.1 (14)	35.3 (6)	25.0 (3)	10.5 (2)	20.0 (3)
**6. Are you interest in attending e-learning training about small-scale dairy processing in the Serbian language?**					
6.1. Yes	67.2 (39)	47.1 (8)	58.3 (7)	84.2 (16)	80.0 (8)
6.2. Maybe	25.9 (15)	52.9 (9)	8.3 (1)	15.8 (3)	20.0 (2)
6.3. No	6.9 (4)	0.0 (0)	33.3 (4)	0.0 (0)	0.0 (0)

Values in the table represent percentages and number - %(N) of respondents giving the positive answer; a–c Values in the superscript within a row with different letters were significantly different (p < 0.05) within clusters, as determined by Mann– Whitney *U* test.

**Fig. 2:**
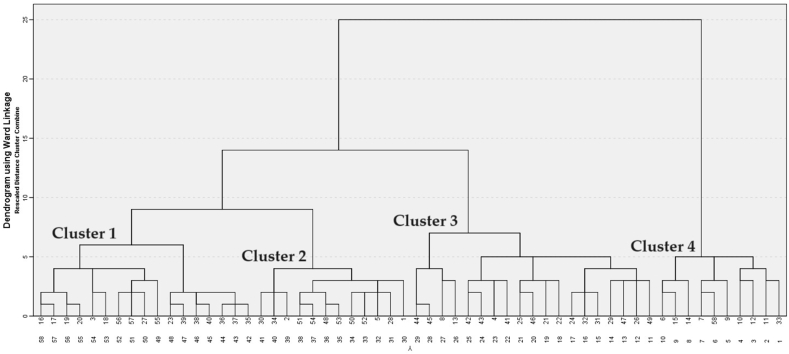
Dendrogram depicting the results of cluster analysis, assigning Small Scale Dairy Processors (SSDPs) to clusters based on their responses to the survey questions.

Hierarchical cluster analysis was applied to all 167 answers from 58 participants. All SSDPs were divided into four clusters using Ward's method and squared Euclidean distance interval for grouping variables (Fig. 2). Nonparametric comparison (Mann-Whitney *U* test) was used to compare the mean sums of responses between clusters. Differences where the *p*-value was less than 0.05 were regarded as statistically significant. The analysis was performed by SPSS Statistics 21 software.

## 3 Results and discussion

### 3.1 Qualitative training needs assessment

The analysis of in-depth interviews with OPRs resulted in seven themes, including: (i) Benefits that OPRs see in working with SSDPs; (ii) Benefits that OPRs perceive SSDPs would have in working with OPRs; (iii) General requirements; (iv) Food safety requirements; (v) Type of SSDPs’ products that fit OPRs target groups, (vi) Challenges and (vii) Areas for improvement.

#### 3.1.1 Theme 1 - benefits that OPRs see in working with SSDPs

DOOSs currently do not offer products from small scale food production. However, they expressed their willingness to do so in the future. The OPRs reported a benefit in promoting local SMEs (small and medium-sized enterprises) and short food chains in terms of social and environmental concerns. They agreed that SSDPs’ products would differentiate them from the traditional large retailers and would align with OPRs target audience – customers with healthy lifestyle and higher income who perceive SSDPs products as healthier than products made by their industrial counterparts, and as authentic, local, “hand-made” products.

#### 3.1.2 Theme 2 - benefits that OPRs perceive for SSDPs in working with OPRs

Both AOP and DOOS's representatives anticipated that by effectively targeting urban and high-paying e-commerce customers, SSDPs would unlock opportunities to sell value-added products, which would positively impact their overall profitability. Participation in the on-line platforms and using their advertising and design services, would provide SSDPs the opportunity to enhance their brand visibility, thereby increasing their popularity and recognition. A small subset of OPRs also provide comprehensive cold chain transportation services, with optimal storage conditions and timely delivery of food products to urban customers. This was recognized as a substantial advantage to SSDPs that would help eliminate logistical challenges, enabling SSDPs to focus primarily on production of superior quality food.

#### 3.1.3 Theme 3 - general requirements for working with online platforms

In collective agreement, the interviewed DOOS and RODO representatives unanimously emphasized that ensuring consistent product quality and uninterrupted delivery from production facilities to e-commerce warehouses are fundamental requirements. In contrast, AOP representatives communicated that their role is limited only to showcasing SSDPs on their platforms and facilitating contact with customers, with legal compliance, transportation, storage, and financial transactions requirements being the responsibility of SSDPs.

#### 3.1.4 Theme 4 - food safety requirements for working with online platforms

Food safety requirements for DOOS and RODO platforms do not differ from the requirements of any other formal retail entity. RODO interviewees indicated that there is no “second-party audit” practice, due to the lack of time and expertise. DOOS representatives asserted that they would unequivocally implement the practice of conducting “second-party audits” when initiating e-commerce with SSDPs, especially for the purpose of hygiene assessment. An AOP representative expressed adherence to such practice for the reason of the utmost prioritization of hygiene when establishing business relationships with SSDPs.

#### 3.1.5 Theme 5 - type of SSDPs' products suitable for OPRs’ target customers

For the purpose of this study, SSDPs products could be classified in two groups: dairy products traditionally consumed in Serbia (e.g., white brined cheese and kajmak) and gourmet dairy products for special occasions (e.g., premium cheeses, mature semi hard/hard cheeses). All interviewees communicated that there is a demand for both types of products, but DOOS and RODO representatives agreed that gourmet dairy products, such as premium mature cheeses are more suitable for their target customers. In addition to the intended use and suitability to the target group of customers, the extended shelf life of mature cheeses was also considered a key factor for this particular product category. In conjunction with the extrinsic product quality, SSDPs were advised to include captivating storytelling and appealing product presentations as additional marketing elements. In contrast, dairy products traditionally consumed in Serbia were estimated to be more suitable for hotels, restaurants and catering sectors.

#### 3.1.6 Theme 6 – perceived challenges in collaboration

The OPRs shared their perceived challenges in working with SSDPs, based on their personal experiences. Collectively, OPRs communicated that SSDPs should be aware of the reputational responsibility once they become members of the e-commerce platform, as the reputation of individual SSDPs affects not only the members of the platform, but also the platform as a whole. To illustrate the reputational risk, an AOP representative highlighted an example of the detrimental impact of fraudulent practices of some SSDPs selling products produced by other SSDPs as their own. They reported that this typically occurs when there is an increase in product demand or when there is a decline in the quality of SSDP's own products. Representatives from the DOOS also acknowledged that the unknown brands pose a challenge within the small-scale dairy product segment, potentially reducing demand for such products. Given that the prices of SSDPs' products are higher compared to mass-produced industrial counterparts with established brands, OPRs acknowledged the need to increase awareness among SSDPs to improve the effectiveness of their branding efforts.

Gender challenges have also been recognized. OPRs reported that generally processing aspects of small-scale dairy operations is carried out by women, while the business aspect is conducted by men, who are not necessarily familiar with dairy processing and products. Gender inequalities in Serbia (especially in rural areas) are underscored by the ‘patriarchal syndrome,’ which confines women to roles of mother and housewife, neglecting their individuality, equality, right to work, and independence [21]. The statement from the RODO representative, indicating that ‘women lack confidence in business,’ aligns with the previously reported issue, underscoring the urgent need for action in this specific field.

#### 3.1.7 Theme 7 - areas for improvement

The interviewed OPRs reported that SSDPs would benefit from the additional knowledge about producing premium dairy products, maintaining consistent product quality, and improving food safety. RODO representative assumed that efforts should be made to educate SSDPs about the benefits associated with product design and presentation. OPRs suggested that SSDPs should be provided with tools and resources to help them achieve food safety and quality targets, as well as practical knowledge and skills. They reported it is crucial to find a solution for the cold chain transportation of SSDPs products from the production sites to the cold storages of e-commerce representatives. To facilitate equipment procurement, logistics, marketing, and various other advantages, OPRs suggested improved networking among SSDPs and establishment of SSDP associations.

### 3.2 Quantitative training needs assessment

SSDPs in Serbia are a highly diverse group, with cluster analysis identifying four distinct clusters. Each cluster is distinguished by unique characteristics. In the following sections, each cluster was characterized, to facilitate the development of tailored training programs that address the distinct challenges, meet the specific needs and focus on the individual strengths and weaknesses of each cluster.

#### 3.2.1 Characterization of clusters – production systems, infrastructure, safety and quality of products and market access

Survey participants’ dairy production systems, processing practices and type of products accross clusters are presented in Table 1 while marketing and distribution practices in Table 2.

Cluster 1 (17 members) – This group of SSDPs is characterized by the production of kajmak (82.4%). Together with kajmak, they also produce white brined cheese from cooked and skimmed milk, as a by-product. Cluster 1 members use kitchen equipment, and thermally treat milk on the stove (100.0%), in large cooking pots. They sell their products in unsealed disposable plastic boxes (82.4%) without labels, mostly at the doorstep (94.1%) to resellers. A high proportion (23.5%) of SSDPs report not being satisfied with the product prices. Not a single processor is a member of an association.

Cluster 2 (12 members) – All members of this cluster produce cheese from raw milk, with products almost exclusively being white brined cheese (58.3%) and fresh cheese (58.3%). They also use kitchen equipment in their production and they tend not to control processing parameters. The products are typically sold in bulk through home-based sales (75.0%) and at open markets (41.7%), with packaging consisting of mainly plastic bags (83.3%) without labels. Half of the producers in this cluster report low product prices as being a major challenge. Many of them reported that they are considering expanding milk production capacity (41.7%) and investing in equipment (58.3%). Three of them (25.0%) are members of associations.

Cluster 3 (19 members) – This cluster produces a mix of products that were listed in the survey, with the majority being producers of white brined cheese (94.7%) and semi hard/hard ripened cheese (68.4%). They use both thermally treated milk (84.2%) and raw milk (52.6%) as the starting material. Only one of the members uses starter cultures in cheese production, but only 15.8% of them process milk in the kitchen. Plant supplements are used by 47.4% of the members. They sell their products in unsealed disposable plastic boxes (78.9%), but a considerable number also use vacuum packaging (63.2%). Only 21.1% have product labels, and less than half of them keep records during production. They participate in all distribution channels but focus more than any other cluster on selling at the open market (63.2%). Most of them reported that there is a lack of suitable market for their products (42.1%), and that they would benefit from the additional education in dairy (31.6%). About half of them (57.9%) are approved by the Veterinary directorate, while less than half of them (42.1%) are members of associations or cooperatives.

Cluster 4 (10 members) – This cluster is mainly focused on the production of semi hard/hard ripened cheese (90.0%), and they also produce specialty cheeses referred to in the questionnaire as “other” cheese (40.0%) (e.g., cheese for grill, *pasta fillata* cheese). They sell their products at food fairs (90.0%) and online platforms (70.0%). They own the basic equipment for cheese making, with most of them pasteurizing milk in cheese vats (90.0%), and 30.0% processing raw milk. Only half of the cluster 4 members use starter cultures, while 70.0% use plant supplements. They keep all types of records. Entire cluster 4 uses vacuum packaging, with labels. They still report the need for the additional education (40.0%) and the challenge they face considering variations in product quality (30.0%). Most of them (70.0%) are approved by Veterinary directorate and are members of associations or cooperatives.

The findings from the qualitative and quantitative analysis revealed a significant gap between the OPR requirements and the conditions practiced by SSDPs, including factors such as proper packaging, labeling, type of product, and cold chain transportation. This is particularly true for members of clusters 1 and 2.

In recent years, growing consumer inclination towards consuming unprocessed food induced the rise in the popularity of local farmers products, artisanal cheeses and raw milk cheese [22]. The results of the current study showed that the entire cluster 2, but also 50.6% of cluster 3 and 30.0% of cluster 4 SSDPs in Serbia produce raw milk cheeses.

Raw milk cheeses have a heterogeneous and diverse microbiota, and thus have richer flavor and a shorter ripening time compared to the pasteurized milk cheeses. At the same time, these types of products have been commonly linked to foodborne illnesses and outbreaks [22], due to their properties being favorable for survival and growth of microbial pathogens, such as *Salmonella* spp., *Listeria monocytogenes*, *Campylobacter* spp., pathogenic *Escherichia coli*, *Mycobacterium* spp., *Brucella* spp., *Staphylococcus aureus*, *Bacillus cereus*, *Clostridium* spp., and others*.* While difficult to attain, strict hygiene both on-farm and during production has been suggested to reduce the prevalence of pathogenic bacteria and help maintain the high quality of raw milk [22]. Based on the data from the current study, the majority of SSDPs are not employing strict hygiene practices, leading to concerns regarding both safety and quality of products made from raw milk.

Specifically, among raw milk cheese producers, only 31.0% of the total number of survey participants reported owning a cooling vat, and 20.7% indicated that they use cleaning and sanitizing products for dairy industry. The entire cluster 1 and 2, but also a substantial part of cluster 3 (73.7%) reported using kitchenware for cheese production. At the same time, not a single member of cluster 2 reported having labels on their products, indicating that customers are often exposed to raw milk products without being aware of it. These findings indicate that there is a need for the development of training resources that focus on food safety risks associated with raw milk cheeses, as well as adequate cleaning and sanitizing practices to reduce food safety risks and improve product quality [23]. At the same time, it would be beneficial to have explicit label declarations indicating products were made from raw milk, to increase consumer awareness when they purchase such products. While EU legislation for food products made with raw milk requires the food label declaration of “made with raw milk” [37], the same requirement is not explicitly stated in the current Serbian legislation [5]. To close this current gap in the Serbian legislation, it would be beneficial to engage policymakers to more clearly define the special hygiene conditions and labeling requirements for raw milk cheese production.

Until recently, taste, price, and convenience were the primary determinants of food customer purchasing decisions. However, buying habits have changed, especially with the emergence of e-commerce. In the present time, food purchasing decisions depend on many factors, including good agricultural practices, food safety, nutritional quality, experience in food purchasing and tasting, transparency, social impact, wellness and health [24]. It has been reported that the majority of on-line food shoppers are concerned with the freshness of products bought on-line [25]. Unlike purchases at the open-market, where customers can visually inspect or even taste products, purchasing through e-commerce is heavily influenced by branding and packaging of food products [25,26]. In the current study 87.9% of SSDP survey respondents indicated that taste is a determining factor for purchasing a product, yet only 34.8% recognized the importance of being able to recognize the product and associated taste through packaging and branding. While less than half of SSDPs surveyed respondents reported having sealed packaging, and only 24.1% reported having a product label, many of them said they are interested in learning about packaging and product marketing (60.3%), and product labeling (48.3%). There seems to be a need for further education on the benefits of investing in brand recognition, including packaging and labeling, and improved advertising through social media platforms.

The majority of SSDPs across all four clusters produce white brined cheese. These producers mostly adhere to a traditional cheese production passed down through generations of family cheesemakers Dozet and Macej [2]. These cheeses are easy to sell due to a high consumer demand [12]. Besides this type of cheese, kajmak is produced by 82.4% and 42.1% of cluster 2 and 3 members, respectively. Kajmak is recognized as a highly esteemed Serbian dairy product obtained as a fat layer collected from the boiled and slowely cooled milk [27]. As a low yield product, producers of kajmak are challenged with making enough product to increase their profits, leading some to adulteration. An alarming observation is that out of the 48 reported instances of food fraud, 17 were associated with kajmak production (unpublished results). The addition of vegetable fats, such as margarine and palm oil, and cellulose powders and flour, are some of the main adulteration techniques reported for this product. These actions constitute not only an economic fraud but also diminish the functional and nutritional value of kajmak and may pose potential health hazards to consumers [28]. To protect the reputation and standards related to kajmak production, and preserve its traditional heritage, it is imperative to raise awareness of kajmak adulteration, and work towards more stringent laboratory testing controls for this product [29].

Semi-hard/hard cheese has been identified by OPRs as the preferred product for their distribution channel, due to its longer shelf life and a potential for being sold at higher prices as premium products. This cheese type is the second most produced cheese variety (46.5%), offered mainly by members of clusters 3 and 4. Considering the prolonged maturation usually associated with the production of semi-hard and hard cheeses, significant inventory and production costs are borne by the cheesemakers for these types of products. In addition, the production of these cheeses requires a higher level of expertise and knowledge due to the multitude of biochemical processes that occur during ripening and require precise control and monitoring [30]. It is therefore important to offer more advanced trainings and skills development for SSDPs interested in expanding their production to semi-hard and hard cheeses.

Aside from the liquid rennet that is available in local markets, SSDPs survey participants reported very low use of other ingredients that are typical in cheese making process. For example, only 10.3% SSDPs reported using starter cultures in their cheese production (e.g., 50.0% of cluster 4 members). Clusters 3 and 4 showed a pronounced tendency towards the utilization of plant supplements (e.g. spices, dry fruits and vegetables). Such supplements serve as essential flavoring agents added to cheese, diversifying its taste and appeal to consumers [31]. Presumably, SSDPs from these two clusters recognized the potential of artisan cheese to be presented to consumers as premium products [32]. All clusters would benefit from additional training on the impact of starter cultures and other process controls to improve product consistency, and ways to add value to their products and production lines in a safe and consistent manner.

#### 3.2.2 Basic digital proficiency and e-commerce perspectives across clusters

The importance of e-commerce development has been an important topic lately, not only because of convenience for customers to access foods and for mitigating negative effects of COVID-19, but also because of the benefits to environment protection and food waste reduction [17]. The majority of SSDP survey participants in the current study demonstrated high level of the basic digital literacy by communicating via e-mail (79.3%) and social media (86.2%), and by using the internet content for self-education (e.g., YouTube tutorials on cheese making (75.9%)). Members of cluster 1 are the least digitally engaged (47.1% have e-mail and 58.8% are on social media). In contrast, 91.7% of cluster 2 and entire cluster 3 and 4 are on Facebook or Instagram (Table 3). However, acquiring additional specific digital competencies is required for SSDP to actively engage in the e-commerce landscape and comprehend the interaction within these platform schemes [33].

Only SSDPs from cluster 3 and 4 use the internet to sell their products (73.7% and 90.0%, respectively), mainly through their own social media profiles (57.9% and 60.0%, respectively). A considerable number of cluster 3 members are also present on AOP platforms (36.8%). Only 8.6% of participants use the option of selling through an online store (RODO) and none sell products through a dedicated online only service (DOOS). The study findings revealed that presently the predominant sales channel for SSDPs is through AOP type. While direct purchases are not possible through this subset of e-commerce, these platforms offer networking, sharing and collaboration between sellers and buyers. On such platforms, buyers’ choices, decisions, and comments generate content that has a significant advertising impact, which can be either very positive or negative in nature [11].

While OPRs reported that participation of SSDPs in e-commerce would allow SSDPs to charge more for their products, access higher-paying customers and ultimately lead to increase in their profits, this was not apparent to many of the SSDPs that participated in the study. Only a small number of SSDPs see the advantage of increasing the price of their products when selling online (18.9%). The primary advantage that SSDPs see in e-commerce is the ability to sell a greater quantity of goods (37.9%), particularly those in cluster 3 (63.2%). When expressing their future plans, they are mostly focused on expanding milk production capacity to increase their profits (e.g., 41.7% of cluster 2 and 42.1% of cluster 3 members). Members of cluster 4 report that they would benefit from e-commerce by being able to sell their products throughout Serbia (60.0%).

Overall, SSDPs do not see many drawbacks in selling through e-commerce. Additional time and organization (34.5%), their own lack of digital skills (10.3%), and the lack of communication with customers (29.3%) are perceived as disadvantages.

Among the e-commerce participants, cluster 3 members transport their products using their own vehicles (31.6%), in hand refrigerators (47.4%), and 31.6% use express shipping services. cluster 4 members are transporting their products in hand refrigerators (70.0%), and 40.0% of them use express shipping services. These findings highlight already reported imperative need for vigilant regulatory oversight in the realm of e-commerce-driven dairy product sales and the necessity of tackling logistical challenges, particularly in response to extended transportation times for packaged portions of dairy products to avoid risk of contamination [34]. As reported, the new and effective transportation and storage solutions is the main area for improvement when e-commerce of food is considered [15].

#### 3.2.3 Training preferences across clusters

There is presently a need to provide SSDPs with more access to information to make informed decisions about joining e-commerce and increasing their profits through different value-added products and production means. This is supported by the data obtained in the current study, which showed that a significant number of SSDPs are interested in participating in training programs tailored to their specific needs. In total, 62.1% of survey participants were interested in attending trainings and 31% expressed conditional interest. Cluster 3 members (84.2%) were the most interested in additional education (Table 4).

While previous research has shown that in-person trainings remain popular among the food industry [35], e-learning online formats are becoming more acceptable due to the location and time flexibility and the lack of financial constrains [36]. With no significant differences between clusters, 75.9% of respondents reported preference for in-person trainings, 41.4% would prefer e-learning training, and more of them are interested if e-courses were offered in their native language 67.2%.

The training topic preferences did not vary between clusters, with the highest interest (79.3%) being the “Improvement of dairy product technology”, while the least preferred (34.5%) topic was “Communication with customers”. Only half of the respondents listed the topic “Food hygiene conditions during production and transportation“ as their training preference (48.3%).

## 4 Conclusion

The results of the present study confirmed that in order to be included in the e-commerce market segment, many SSDPs from Serbia require both product and production adjustments. To do this effectively, it is essential to train all SSDPs in safe food production and distribution and provide them with knowledge and skills to produce safe food products with longer shelf life, high intrinsic and extrinsic quality, adequate packaging and proper labels. Such products would be more competitive in both traditional dairy products and e-commerce markets. By analyzing the differences among clusters of SSDPs trainings can be tailored to the characteristics of each group, to optimize their learning experience and enhance their overall productivity, efficiency, and market success.

SSDPs that want to enter e-commerce should adopt a proactive and open-minded attitude toward internet selling and learning. Firstly, they should embrace the transformative potential of e-commerce, and recognize it as an opportunity to expand their market reach, and enhance profitability. Understanding the unique advantages that online platforms offer, such as broader customer access, convenience, and cost-effectiveness, is essential in cultivating a positive attitude. By continually educating themselves and staying updated with emerging trends in e-commerce, SSDPs can adapt to the evolving digital landscape and remain competitive.

The identification of knowledge gaps and specific training requirements for small scale dairy processors contributes to the development of targeted training programs. This is particularly beneficial for enhancing the capabilities of small scale dairy processors in aspects such as food safety, product quality, and online marketing. The cluster analysis results enhance the study's applicability by allowing for tailored interventions.

The findings of the current study underscore the need for targeted training programs for SSDPs, focusing on providing them with the tools, resources, and knowledge required to achieve food safety standards. This includes practical skills and awareness related to maintaining the quality and safety of dairy products. Compliance with food safety regulations and quality standards is likely to be a prerequisite for successfully marketing and selling dairy products online. The research points out the importance of finding solutions for cold chain transportation to ensure consistent product quality but also for meeting food safety standards. Improving labeling practices could contribute to better transparency and consumer confidence in the safety of the dairy products.

While many findings from this study can likely be applied to SSDPs in other regions, especially in the Balkans area, it is important to note that the extent of comparison is limited because existing infrastructures, standards and regulations differ across countries, which are likely to influence how SSDPs operate and access e-commerce. Nevertheless, this study can serve as a basis for other research focused on assessing needs for small-scale processors in accessing e-commerce in different geographical areas and understanding challenges and gaps to better tailor resources and educational initiatives.

## Funding

This work was supported by the 10.13039/100000200United States Agency for International Development (USAID) [agreement number: AID-OAA-A-11-00012; grant number: COV-183 Serbia] as a part of the project “Mitigating effects of COVID-19 on small-scale dairy farmhouse producers through education and training initiatives to improve food safety and their access to online distribution platforms” included in the Partnerships for Enhanced Engagement in Research (PEER) program.

## Data availability statement

Data will be made available on request.

## CRediT authorship contribution statement

**Zorana Miloradovic:** Writing – original draft, Project administration, Methodology, Investigation, Conceptualization. **Jovana Kovacevic:** Writing – review & editing, Conceptualization. **Jelena Miocionovic:** Writing – review & editing, Investigation. **Ilija Djekic:** Writing – review & editing, Investigation. **Nemanja Kljajevic:** Writing – review & editing, Formal analysis. **Nada Smigic:** Writing – review & editing, Supervision, Project administration, Investigation, Funding acquisition, Conceptualization.

## Declaration of competing interest

The authors declare that they have no known competing financial interests or personal relationships that could have appeared to influence the work reported in this paper.
