# Supplementary material for: E-commerce readiness and training needs of small-scale dairy processors in Serbia: Understanding barriers and knowledge gaps
Source: Heliyon. 2024 Mar 7;10(6):e27442. doi: 10.1016/j.heliyon.2024.e27442 (PMC10945173; doi:10.1016/j.heliyon.2024.e27442)
Supplement: Multimedia component 1 [file mmc1.pdf]

Code book

Themes identified during in-depth interviews with the online platforms representative (n=7)

**Themes**	**Codes**	**Quotes from the interviews**
Benefits that OPRs see in working with SSDPs	Digitalization of SME	“We are socially responsible company. One of our directions is to give small and medium-sized businesses (SME) a chance in digitization and bring them closer to the market.” (male, 25-34 years, DOOS)
	Environmental issues	“Our tendency is to promote short chains in food distribution, for the environmental issues.” (male, 25-34 years, DOOS)
	The image of the company	“Working with SSDPs would make a great story for public image of our company.” (male, 35-44 years, DOOS)
	Special characteristics of SSDP products	“SSDP products are perceived as healthy, hand-made, local.” (male, 25-34 years, DOOS)
	Differentiating from hypermarkets	“Having SSDP products in our assortment would differentiate us from supermarkets and hypermarkets that are focused on the industrial food products.” (male, 35-44 years, DOOS)
	Fitting to e-commerce target group	“We estimate that there would be a demand for high price, premium SSDP cheeses.” (male, 35-44 years, DOOS)<br>“Customers with higher socio-economic status are a target group for our food e-commerce business.” (male, 35-44 years AOP)
Benefits that OPRs perceive for SSDPs in working with OPRs	Reaching city customers	“Premium SSDP cheeses would have their customers only in the capital city and a few other large cities in Serbia.” (male, 35-44 years, DOOS)
	Gaining visibility	“The purpose of our association is to make a connection between producers from villages with customers from cities.” (female, 35-44 years, AOP)<br>“SSDP would gain high visibility by being members of our platform.” (male, 35-44 years, DOOS)<br>“For SSDP, our platform is also a space for advertising their products and telling their stories.” (female, 35-44 years, AOP)
	Value added products	“At the moment, majority of SSDPs do not perceive their products as value added food that could be in demand by e-commerce customers.” (female, 35-44 years, AOP)<br>“SSDP have to be aware that their future is in producing value added products, especially Kajmak, which is our best known dairy product – very expensive to produce.” (male, 35-44 years, AOP)
	Logistics	“We have storage with cooling chambers, and cold-chain food delivery.” (male, 25-34 years, DOOS)<br>“There is a limited number of SSDPs who have their own delivery service platform, and also not many of them are delivering products to customers on their own.” (female, 35-44 years, AOP)<br>“SSDPs would be able to deliver a large number of their products to the customers, and then they do not have to think about anything but safety and quality of their products.” (male, 35-44 years, AOP)
	Design	“Whenever our designer improves or adds something to our platform, the sales of SSDP products increase.” (female, 35-44 years, AOP)

Themes	Codes	Quotes from the interviews
General requirements for working with online platforms	Basic requirements	“To be registered as a household or a company; to be registered/approved by the Veterinary directorate for the production of dairy products, and to have an adequate packaging/declaration.” (female, 35-44 years, RODO)<br>“Anything that is needed for any other distribution channel is also needed for e-commerce through our platform.” (male, 25-34 years, DOOS)
	Continuity	“Continuity in the delivery and consistent quality.” (male 35-44 years, RODO)<br>“Unified mass of each product item (often hard to achieve in artisan product manufacturing).” (male, 35-44 years, DOOS)
Food safety requirements for working with online platforms	Second party audit	“Second party audit would be conducted once we add SSDPs to our platform.” (male, 25-34 years, DOOS)<br>“I do the second party audit, because hygiene is what I value the most, for my decision to collaborate with SSDPs.” (male, 35-44 years, AOP).
	Cold chain	“We do not check the temperature of the transportation vehicle as long as we are satisfied with the product.” (male, 35-44 years, RODO).
	Spoilage/ Expiration date/recalls	“It happens occasionally that the mold appears before the expiration date. In such cases the product is returned to the producer.” (male, 35-44 years, RODO).<br>“SSDPs usually give the shorter expiration date than needed, just in case.” (female, 35-44 years, RODO)
	Personal responsibility	“SSDPs care a lot about their business, because it is their name on the product.” (female, 35-44 years, RODO)
Type of SSDPs’ products suitable for OPRs' target customers	Commodity products	“Eating habits are dictating the type of the products in demand. White brined cheese and Kajmak are the only option for customers in central Serbia for example.” (female, 35-44 years, RODO)
	Premium products	“For Belgrade customers and our platform - just premium products! The ones that could be offered together with wine, dry meat products, etc.” (male, 35-44, DOOS)
	Mature cheese	“For our unstable market, a product with long shelf life (such as mature cheese) would be a good product for SSDPs’ production.” (female, 35-44 years, RODO)
	Healthy life style	“Products associated with healthy life style are what our customers increasingly demand.” (male, 25-44 years, DOOS)
	Storytelling/brand	“Customers love to hear the stories behind cheese production. And they like to recognize the brand.” (female, 35-44 years, RODO)
	Product presentation	“Customers love and want to try the product that is well presented (e.g., handmade, special, with a story).” (male, 35-44 years, RODO)
Perceived challenges in collaboration	Collective responsibility	“A single producer can put the reputation of the entire online platform at risk with a drop in product quality, unprofessional communication, and/or adulteration of the product.” (female, 35-44 years, AOP; male 35-44 years, AOP)
	Gender challenges	“Households and business aspects are officially run by men and manufacturing is done by women, who often lack confidence in doing business themselves.” (female, 35-44 years, RODO)
	Age challenges	“Many SSDP manufacturers are age 50 and above, who are not familiar with digital tools and have resistance towards e-

Themes	Codes	Quotes from the interviews
		commerce .” (female, 35-44 years, RODO)
	Fragmented production	“Regarding paperwork, it is complicated to have many suppliers with only one product each – which is often the case for SSDPs.” (male, 35-44 years, DOOS)
	Unknown brands	“Unknown brands of SSDPs are a challenge. Customers prefer famous brands, except for the adventurous high-paying customers who may take more risks for the new experiences.” (male, 35-44 years, DOOS)
	Industrial products as competition	“The industrial products of the similar type are competitive in price in comparison to SSDPs products.” (male, 25-34 years, DOOS)
	Unawareness of their own fraudulent activities	“When the demand increases, selling products produced by other SSDPs as your own, or the decrease in quality of products is often being practiced among SSDPs.” (female, 35-44 years, AOP)
Areas for improvement	Product wholeness	“Wholeness of the product, including packaging, design, and branding, is needed, and for SSDPs to learn about the benefits this would bring to them.” (male, 25-34 years, DOOS)
	Registration	“They all need to get registered/approved by the Veterinary directorate.” (female, 35-44 years, RODO)
	Quality	“Matured cheese produced in SSDPs vary in quality. SSDPs lack knowledge in premium cheese production.” (female, 35-44 years, RODO)
	Packaging and design	“White brined cheese and kajmak need modern, well designed packaging.” (female, 35-44 years, RODO)<br>“Better presentation of the product is needed – to which target group they offer it, how it is served.“ (male, 35-44 years, RODO)
	Transportation and storage	“Finding cold storage options in Belgrade.” (female, 35-44 years, RODO)<br>“Engaging professional food carriers with cooling vehicles, or investing in own cooling vehicles.” (female, 35-44 years, RODO)
	Network	“Peer-to-peer education.” (male, 25-34 years, DOOS)<br>“Connecting with other small food producers of premium products such as wine or fruit spreads to learn about market opportunities.” (male, 35-44 years, DOOS)
	SSDPs Associations	“For the purpose of the purchasing equipment, logistics and marketing, developing an SSDP associations could help with many challenges that SSDPs are facing at the moment.” (male, 35-44 years, AOP)
