# Supplementary material for: E-commerce readiness and training needs of small-scale dairy processors in Serbia: Understanding barriers and knowledge gaps
Source: Heliyon. 2024 Mar 7;10(6):e27442. doi: 10.1016/j.heliyon.2024.e27442 (PMC10945173; doi:10.1016/j.heliyon.2024.e27442)
Supplement: Multimedia component 2 [file mmc2.pdf]

Dear respondents, please read the following information carefully before proceeding

The questions you are about to answer are designed to assess the needs for training among small scale dairy producers in Serbia. Your answers, along with the answers of other participants, will be grouped and used to determine areas of training that can be improved.

Your participation in the study is completely voluntary. You can choose not to participate or not to answer any specific questions. If you choose not to participate or answer, there will be no penalty or loss of benefits to you.

Your information will be kept confidential to the researchers. Your individual responses and information will not be shared with anyone outside of the research team. Your information will be added to responses from other participants. The will be no identifying information released which would allow someone to identify your answers.

Thank you

Processing practices and type of products

1. Have you been registered as an agricultural household?
    a. Yes
    b. No
2. Have you been registered by the Veterinary Directorate?
    a. Yes
    b. No, but we are currently in the registration process
    c. No, but we are planning to apply for registration
    d. No
3. Have you been approved by the Veterinary Directorate?
    a. Yes
    b. No, but we are currently in the process of approval
    c. No, but we are planning to apply for approval
    d. No
4. Are you a member of an association/cooperative?
    a. Yes
    b. No
5. What milk products do you produce (multiple choices possible)?
    a. White brined cheese
    b. Cream cheese/Quark type cheese
    c. Whey cheese
    d. Whey
    e. Semi hard/hard cheese
    f. Kajmak
    g. Other
6. Where do you get raw milk?
    a. I only use raw milk from my own farm
    b. I use raw milk from my farm, but I also get it from my neighbors
    c. I use raw milk that I get from my neighbors
7. Is the volume of the milk you process in complience with the regulation?
    a. Yes
    b. No
8. How do you perform milking of the animals at your farm?
    c. Manually
    d. Mechanically
    e. Combined, manually/mechanically

9. Where do you process raw milk (multiple choices possible)?
    a. In the kitchen
    b. In a separate room of the house
    c. In a separate building in the yard of the house
10. What type of milk do you use to make cheese (multiple choices possible)?
    a. Boiled/thermally treated milk
    b. Uncooked/raw milk
11. Which of the listed ingredients do you use in the production of cheese (multiple choices possible)?
    a. Liquid rennet
    b. Rennet powder
    c. Starter culture
    d. Acids (e.g. lactic, citric, acetic)
    e. Food colors
    f. Plant supplements (e.g. rosemary, paprika, cranberries)
12. Which of the listed equipment do you use in milk processing (multiple choices possible)?
    a. Pot, kitchen table, plastic dishes and stove
    b. Cooling vat
    c. Cheese making vat
    d. Cheese press
    e. Cheese molds
    f. Cheese table
13. During milk processing, which parameters do you control (multiple choices possible)?
    a. Temperature
    b. Time
    c. Acidity
14. What cleaning and disinfecting agents do you use during milk processing (multiple choices possible)?
    a. Kithcen cleaning products (e.g. Mer, Fairy, Cif)
    b. Dairy industry cleaning products (e.g. oksimon, alkopen, acidopen)
    c. Other
15. How do you package your dairy products (multiple choices possible)?
    a. Plastic, disposable box
    b. Plastic, sealed box
    c. Plastic bag
    d. Vacuum package
    e. Wax
    f. Other
16. Do you have a product label on the packaging?
    a. Yes
    b. No
17. If you have a product label, what does it contain (multiple choice possible)?
    a. Product name
    b. List of ingredients
    c. Production date
    d. Expiration date
    e. Storage conditions
    f. Manufacturer's name and address
    g. Registration number of the object
    h. Weight/mass
    i. Logo/Some special image/graphic

j. Special labels (organic, protection of origin, etc.)

18. What records do you keep during milk processing (multiple choices possible)?
    a. Dairy processing journal
    b. Records on the procurement of materials and ingredients
    c. Record of sold goods

Marketing and distribution practices across clusters

19. Where do you sell your products (multiple choices possible)?
    a. Home-based (on your doorstep)
    b. In your own catering facility /rural tourism
    c. At the open market
    d. At a food fair/ festival
    e. In a retail store
    f. Via internet/online platform
    g. In a restaurant, hotel, catering
20. Have you ever returned unsold products back to the facility?
    a. Yes
    b. No
21. What did you do with that product if it was within the expiration date (multiple choices possible)?
    a. We eat it
    b. We sell it as soon as possible
    c. We throw it in the garbage
    d. Pigs eat it
    e. Other
22. In your opinion, what is the most important thing for selling dairy products (multiple choices possible)?
    a. Product appearance
    b. The taste of the product
    c. The price
    d. Packaging
    e. Communication with customers
    f. Other
23. If anyone would complain about the quality of the product, how would you react (multiple choices possible)?
    a. I would apologize
    b. I would give a discount on the next purchase
    c. I would offer a similar product for free
    d. I would return customers money
    e. Nothing, I can’t please everyone
    f. Other
24. Have you heard of the term "food fraud"?
    a. Yes
    b. No
25. How many instances of food fraud can you remember?
    a. _______________
    b. _______________
    c. _______________
26. In your opinion, what is "food fraud" (multiple choices possible)?
    a. Selling milk from cows that were given antibiotics
    b. When selling a neighbors’ product claiming it’s ours

c. When milk is diluted with water
d. When the product is sold as fresh, and it was made 10 days ago
e. Other

27. What are your plans for the future (multiple choices possible)?
    f. Expanding the assortment of products
    g. Expanding the capacity of milk production
    h. Investing in the equipment
    i. Cessation of production
    j. Other
28. What are your major challenges (multiple choices possible)?
    k. Product spoilage
    l. Variations in product quality
    m. Low price of the product
    n. Lack of an market
    o. Lack of knowledge
    p. Other

Proficiencies and e-commerce perspectives

29. Do you have an e-mail account?
    h. Yes
    i. No
30. If you do, how often do you check your e-mail?
    a. At least once a day
    b. Once a week
    c. When it comes to my mind, rarely
31. Do you have Facebook/Instagram profile/account?
    j. Yes
    k. No
32. If you have a Facebook/Instagram profile, how often do you check them?
    a. At least once a day
    b. Once a week
    c. When it comes to my mind, rarely
33. Do you use the Internet to sell your products?
    a. Yes
    b. No, but I have planned to use it
    c. No
34. If you use the Internet to sell your products, how do you do it (multiple choices possible)?
    a. I have my own website
    b. I have my Facebook/Instagram profile
    c. I sell through association with an online platform
    d. I sell through the retail with an online sales option
    e. I sell through dedicated online only service
35. If you use the Internet to sell your products, how do you deliver them (multiple choices possible)?
    a. By own vehicle, in the trunk
    b. By own vehicle, in hand-held refrigerators
    c. By own vehicle, which has a cooling chamber
    d. I use express mail services
    e. I am hiring a transporter with a refrigerated vehicle
36. In your opinion, what are the advantages of selling on the Internet (multiple choices possible)?

a. I can sell goods faster
b. I can sell more goods
c. I can sell all over Serbia
d. I can communicate with customers faster
e. I can charge immediately
f. I can charge more for products
g. Other

37. In your opinion, what are the disadvantages of selling on the Internet (multiple choices possible)?
    a. I have no direct communication with the customer
    b. I don't have enough knowledge
    c. I can't be on the Internet all the time
    d. I don't like payment via account, I like cash
    e. I don't want others to make money off of me
    f. Additional time and additional organization are necessary
    g. Other

Training preferences

38. Have you attended any training related to milk processing and businessIn the last five years?
    a. Yes
    b. No
39. If training on milk processing is organized on farms, would you be interested in attending such training?
    a. Yes
    b. Maybe
    c. No
40. If you are interested in attending the training, which way of conducting the training would be the best for you?
    a. Face-to-face
    b. Via internet/e-learning training
    c. Other
41. If you are interested in attending the training, what topics would you choose (mark all that apply)?
    a. Legal requirements and regulations
    b. Communication with customers
    c. Improvement of dairy product technology
    d. Production, packaging and product marketing
    e. Food hygiene conditions during production and transport
    f. Product labeling
    g. Other
42. Have you ever watched content related to milk processing on the Internet?
    a. Yes, I attended online training
    b. Yes, I watched various you-tube contents
    c. No
43. If there were trainings on the Internet about small scale dairy processing in the Serbian language, would you be interested in attending such a training?
    a. Yes
    b. Maybe
    c. No
